# Effect of Microstructure on High-Speed Tensile Mechanical Properties of Ti-1300 Alloy

**DOI:** 10.3390/ma16134725

**Published:** 2023-06-29

**Authors:** Zhu-Ye Zhang, Dong-Rong Liu, Zhen-Peng Pu

**Affiliations:** 1 School of Materials Science and Chemical Engineering, Harbin University of Science and Technology, No. 4 Lin Yuan Road, Harbin 150040, China; zhangzhuye@tongji.edu.cn (Z.-Y.Z.); 2210203038@stu.hrbust.edu.cn (Z.-P.P.); 2 Key Laboratory of Advanced Manufacturing and Intelligent Technology, Ministry of Education, School of Mechanical Power, Harbin University of Science and Technology, Harbin 150000, China

**Keywords:** metastable β titanium alloy, microstructure, solution treatment, high-speed tensile

## Abstract

It is usually required that Ti-1300 alloys be able to withstand a greater load under special conditions, such as the controllable collision of a space shuttle and rapid collision of an automobile. Because of a good combination of strength and toughness, Ti-1300 alloys are widely applied in the aerospace industry. However, during the service process, the alloy components inevitably bear extreme loads. This paper uses high-speed tensile technology to systematically study the effects of different strain rates on the deformation of the microstructure and deformation mechanism of Ti-1300 alloys and to clarify a relation between the microstructure and mechanical properties. The results show that no phase transformation occurs during the high-speed tensile process at strain rates of 200 s^−1^ and 500 s^−1^. The deformation mechanism is mainly due to dislocation slip. The fracture mode is ductile fracture at the two strain rates, due to the connection between micro-voids promoted by dislocation slip. The ultimate tensile strengths are 1227 MPa and 1368 MPa, the yield strengths are 1050 MPa and 1220 MPa, and the elongations are 11.3% and 10.4%, respectively. The present results provide theoretical guidance for the further application of metastable β titanium alloys in working environments with high strain rates.

## 1. Introduction

Titanium alloys are widely used in aerospace, marine, automotive, and biomedical fields because of their high strength, high temperature resistance, corrosion resistance, non-toxicity, and good weldability [1,2]. With the development of the aerospace, marine and automotive industries in recent years, the requirements for excellent mechanical properties, such as a high strength, high toughness and fatigue resistance, are becoming urgent. 

Metastable β titanium alloys have a high strength (UTS ≥ 1100 MPa) and high toughness (Elongation ≥ 6%), which are largely dependent on precise thermo-mechanical processing and proper solution treatment. At present, there are many titanium alloys that are denoted as metastable β titanium alloys, including Ti-5553, Ti-1023 and Ti-1300. Among them, Ti-1300 alloys show a good combination of strength and toughness. Therefore, Ti-1300 alloys have gradually attracted researchers’ attention [3,4].

Ti-1300 alloys, as metastable β titanium alloys, belong to a six-component system with molybdenum equivalent [Mo] = 12.8 [5]. Lu et al. systematically studied the microstructure and tensile properties of Ti-1300 alloy by controlling the precipitation behavior of α-phase [6,7]. The obtained tensile strength and yield strength were 1350 MPa and 1000 MPa, respectively, and the elongation was 13.5%. In Refs. [8,9], forging β phase can improve mechanical properties; for example, the fracture toughness was raised to be higher than 50 MPa·m^l/2^. Thus, forging has become an important way to produce titanium alloys [10]. 

The microstructure of Ti-1300 alloys consists of (α + β) dual-phase, where the single β phase is mainly achieved by solution treatment. The single β equiaxial microstructure performs better in terms of the impact toughness, fatigue limit, and plastic deformation. Meanwhile, the room-temperature deformation of Ti-1300 alloys is mainly influenced by β-phase stability, grain size, strain rate, and chemical composition [11,12,13,14]. Among these factors, the strain rate has the greatest influence [15]. Wan et al. [16] studied the effects of solution treatment on the room-temperature deformation and mechanical properties of Ti-1300 alloy using quasi-static stretching (1 mm/min). The results showed that the tensile strength and yield strength increased with an increasing solution-treatment temperature but that the elongation presented opposite trends. The room-temperature deformation mechanism was dislocation slip. Subsequently, the thermal compression behavior of Ti-1300 alloy at different strain rates (0.01–10 s^−1^) was investigated in [17,18]. The results showed that the flow stress was related to the strain rate and solution-treatment temperature. The thermal deformation mechanism was mainly dynamic recovery and dynamic recrystallization. To better investigate the deformation mechanism of titanium alloys at higher strain rates, Tayyeb Ali et al. [19] studied the quasi-static compression and dynamic compression of Ti-5553 alloy using strain rates of 10^−3^~10^3^ s^−1^. The main deformation mechanisms of quasi-static compression and dynamic compression were dislocation slip and twinning. Chen et al. [20] investigated the dynamic compression mechanical behavior of two-phase (α + β) Ti-1023 alloy at 2000~4000 s^−1^ using the split Hopkinson pressure bar, which showed that the dynamic yield strength and ultimate strength were on the rise and that the plasticity decreased. The deformation mechanism was mainly twinning.

Therefore, to ensure that key structural components of large passenger aircraft have both a high strength and toughness under various dynamic loads, it is necessary to study and estimate the dynamic mechanical properties of Ti-1300 alloys by means of high-speed tensile experiments [21,22,23,24,25].

This paper systematically studies the microstructural deformation and deformation mechanism of Ti-1300 alloy by high-speed tensile testing on the basis of solution treatment. Investigations on the microstructure evolution, phase transformation and various crystal defects are performed. 

## 2. Experiment Section

### 2.1. Experimental Materials and Solution Treatment

The forging state of Ti-1300 titanium alloy (Nominal concentration, Ti-5Al-4Mo-4V-3Zr-4Cr, wt.%) sheet (length × width × height: 113 mm × 22 mm × 18 mm) was used in this study. It was provided by the Northwest Institute For Non-ferrous Metal Research.

The phase transformation temperature from (α + β) phase to single phase is *T_β_* = 830 ± 5 °C [8,26]. A muffle furnace was used with a heating rate of 300 °C/h. The maximum furnace temperature was 1200 °C. Vacuum treatment was not required. To better understand the deformation mechanism of Ti-1300 alloy under high-speed tensile testing, it is necessary to change the initial (α + β) microstructure to a single β microstructure to establish a relationship between the microstructure and mechanical properties. Therefore, the solution treatment is carried out with a temperature of 870 °C and holding time of 2.5 h. 

To prevent alloy oxidation, the Ti-1300 alloy sheet samples within quartz were sealed by a flame spray gun and were then placed in the muffle furnace. After taking out the samples from the furnace, the samples were quickly placed into cold water for water quenching. There were no potential sources of contamination during the whole process. 

### 2.2. Microstructure

In order to observe the deformed microstructure at strain rates of 200 s^−1^ and 500 s^−1^, the sample was cut into a size of 8 mm × 4 mm × 2 mm along the tensile direction. The testing samples were prepared by grinding, mechanical polishing and electrolytic polishing. The mechanical polishing agent was 0.3 μm diamond polishing powder with OPS liquid. The chemical solution was 3 mL HF + 5 mL HNO_3_ + 92 mL H_2_O. After corrosion for 30 s, the metallographic data were obtained by the Carl Zeiss Axio Observer optical microscope. The electrolytic polishing solution was 2 mL perchloric acid + 11 mL n-butanol + 20 mL methanol. The high-speed tensile fracture morphology at 200 s^−1^ and 500 s^−1^ was photographed using a SU7000 Schottky field emission scanning electron microscope (SEM). 

The EBSD measurement was performed on the Oxford Electron Backscatter Diffraction (EBSD) detector. The step size was 3.5 μm, and the scanning area was 2.5 mm × 2.5 mm. Deformation of Ti-1300 alloy under high-speed tensile testing was observed using a Talos F200S field emission transmission electron microscope (TEM). The required specimens with a thickness of about 30~50 μm were made by sandpaper grinding. Then, ion-beam thinning was used to prepare the TEM samples. The working voltage was set to 20~30 V. The current was set to 30~50 Ma. Liquid nitrogen was used to cool the specimens to −25~−35 °C. XRD (X-ray Diffraction) was performed through an X ‘Pert PRO X-ray diffractometer. 

### 2.3. Mechanical Properties

**Figure 1:**
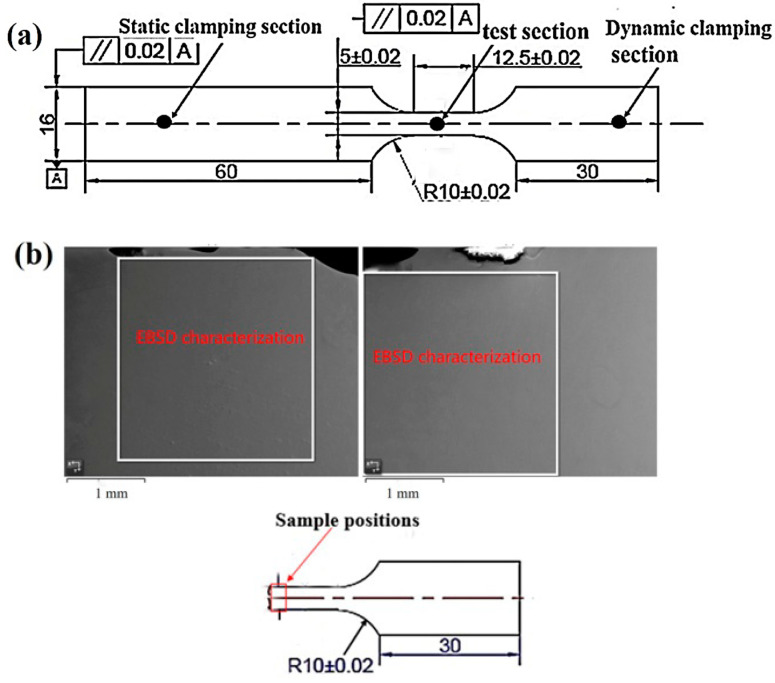
Schematic of (**a**) Ti-1300 high-speed tensile test sample; (**b**) Position (white square) for experimental characterization.

The sheet with solution-treatment parameters of 870 °C/2.5 h was machined into a high-speed tensile specimen (length × width × height: 112 mm × 16 mm × 2 mm). The high-speed tensile samples of Ti-1300 alloy consisted of three parts, a static clamping section, dynamic clamping section and test section (Figure 1a). The position of the sample used for experimental characterization is shown in Figure 1b. The INSTRON VHS 160/100-20 universal hydraulic servo tester was used to conduct a high-speed tensile test at strain rates of 200–500 s^−1^. The experimental parameters are shown in Table 1.

## 3. Results and Discussion

### 3.1. Microstructure of Ti-1300 Alloy after Solution Treatment

**Figure 2:**
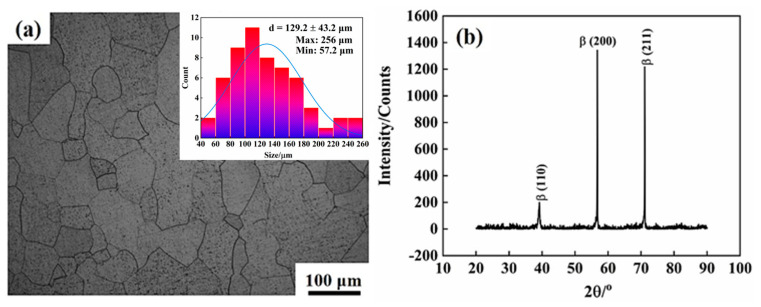
Ti-1300 alloy after solution treatment at 870 °C/2.5 h: (**a**) Metallographic image; (**b**) Phase structure.

Figure 2 shows the microstructure image and XRD result of Ti-1300 alloy after solution treatment. One can see that without other phase diffraction peaks, only β phase exists. The strong diffraction peaks of β phase are β (200) and β (211), respectively (Figure 2b). The diffraction peaks of β phase are narrow and sharp, indicating that the grain size of single β phase is large. The average grain size is 129.2 μm, the maximum grain size is 256 μm, and the minimum grain size is 57.2 μm (Figure 2a). 

### 3.2. Microstructure

#### 3.2.1. Phase Structure of Ti-1300 Alloy

In order to study the effects of strain rates of 200 s^−1^ and 500 s^−1^ on the tensile deformation of Ti-1300 alloy at room temperature, the tensile deformation area was analyzed by XRD.

**Figure 3:**
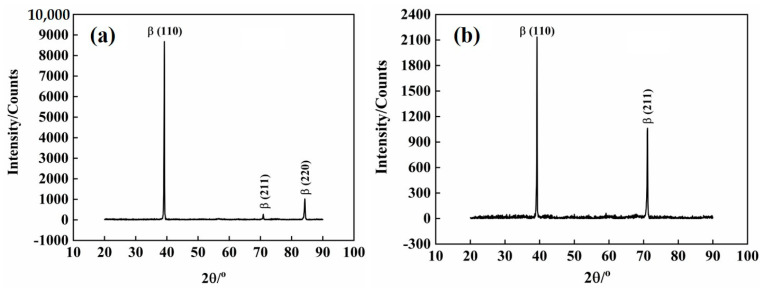
Effects of strain rates on the phase structure of Ti−1300 alloy: (**a**) 200 s^−1^ and (**b**) 500 s^−1^.

From Figure 3, it can be seen that there is only β phase. No α phase or other phase diffraction peaks are found. At strain rates of 200 s^−1^ and 500 s^−1^, the phase structure of the alloy was not affected. Because of the high equivalent molybdenum of Ti-1300 alloy, the stability of β phase is good. It is not easy to produce stress–strain-induced martensite. 

At the same time, with the increase of strain rates, the diffraction intensity of β phase varies. The main peak β (110) peak is basically unchanged, the second strong peak β (211) peak is significantly increased, and the second strong peak β (220) peak disappears. It can be seen that the strain rate affects the intensity of the diffraction peak. This may be due to the different microstructures formed under different deformation conditions. The different microstructures have different preferred orientations.

#### 3.2.2. Analysis of Deformation Microstructure of Ti-1300 Alloy

**Figure 4:**
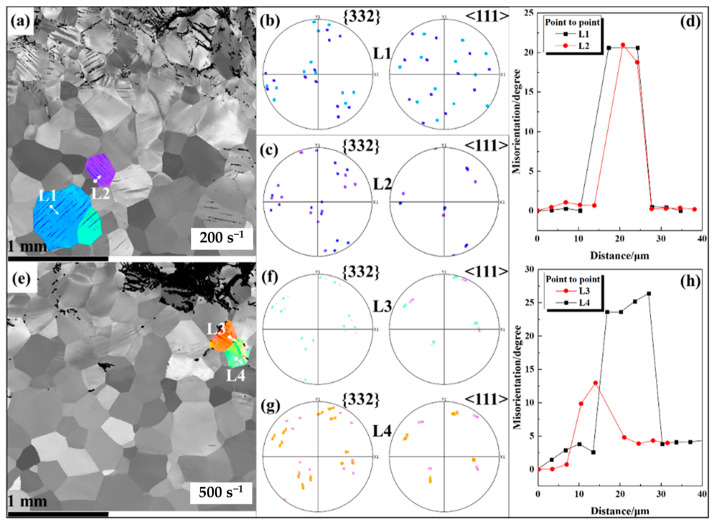
(**a**) IPF map with a subset of two characteristic grains at a strain rate of 200 s^−1^; (**b**,**c**) the {332} lattice plane and <112> lattice direction pole figures for L1 and L2 grains, respectively; (**d**) the line distribution of misorientation for L1 and L2; (**e**) IPF map with a subset of the characteristic grains at a strain rate of 500 s^−1^; (**f**,**g**) the {332} lattice plane and <112> lattice direction pole figures for L3 and L4 grains, respectively; and (**h**) the line distribution of misorientation for L3 and L4.

The XRD analysis of the fracture area shows that no phase transformation occurred during the dynamic loading (Figure 3). Thus, a single β-phase microstructure was obtained. A needle-like structure with a parallel-distribution pattern was observed within the grains (Figure 4a,e), which was preliminarily determined as a possible twinning of β phase. Therefore, two grains containing the needle-like structure were selected to further analyze their characteristics. The two grains are L1 and L2 at a strain rate of 200 s^−1^ (Figure 4a). The possible twinning system was analyzed using a pole figure with a line distribution of misorientation. The pole figures corresponding to the {332} lattice plane and the <112> lattice direction of the two grains are shown in Figure 4b,c, respectively. No mirror symmetry relationship was observed between the needle-like structure and matrix on the twinning plane and along the twinning direction. Furthermore, the results of the line misorientation distribution for L1 and L2 (Figure 4d) showed that the misorientation between the matrix and needle-like structure was about 21°, which largely deviated from the theoretical value of 50.5° corresponding to the β-phase {332} <111> twins [27,28]. The substructure within the grains at a strain rate of 200 s^−1^ is therefore not a twin but a slip band. The same method was used to analyze the characteristics of the grains L3 and L4 at a strain rate of 500 s^−1^ (Figure 4e). The results are similar to those at 200 s^−1^. The mirror symmetry of the needle-like structure with respect to the matrix was not observed in the pole figure (Figure 4f,g). The misorientation also differs from the theoretical value (Figure 4h), so that the needle-like structure is also a slip band.

**Figure 5:**
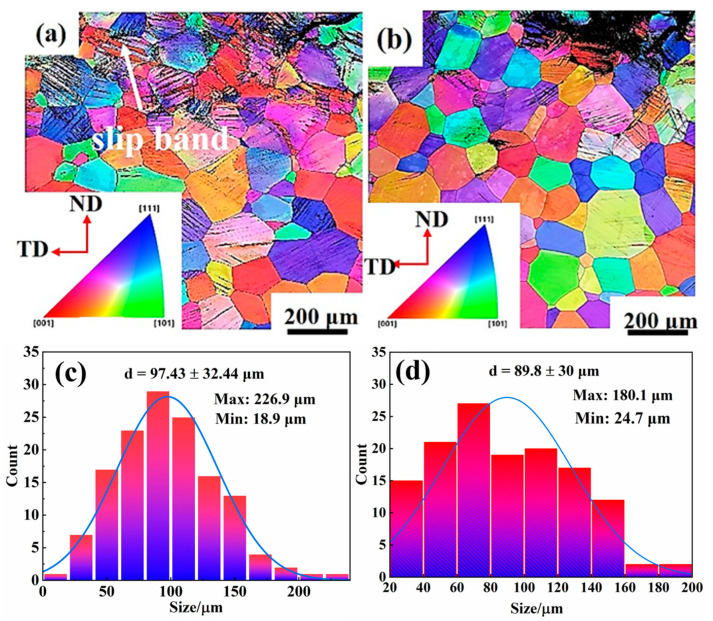
The inverse pole figure (IPF) of Ti−1300 alloy deformed at strain rates of (**a**) 200 s^−1^ and (**b**) 500 s^−1^. Histogram of grain size at strain rates of (**c**) 200 s^−1^ and (**d**) 500 s^−1^.

The EBSD characterizations of the grain structure near the fracture at the two strain rates of 200 s^−1^ and 500 s^−1^ are shown in Figure 5. Because the severe deformation is close to the necking, the resolution of IPF maps is limited. The parallel distribution of the needle structure inside the grains is observed in the IPF maps, representing slip bands. It should be stressed that although the two EBSD maps look similar to each other, the occurrence of slip bands near the fracture is more obvious at 500 s^−1^. It seems that the localized deformation exists at 500 s^−1^ (Figure 5b). However, this explanation needs further analysis. Moreover, the histograms of grain sizes at the two strain rates are similar (Figure 5c,d). No refinement of grain structure is noted during high-speed tensile testing.

**Figure 6:**
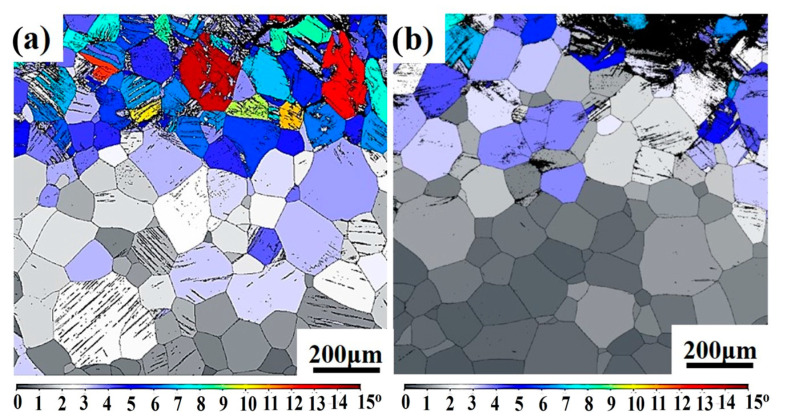
Grain orientation spread (GOS) maps at strain rates of (**a**) 200 s^−1^ and (**b**) 500 s^−1^.

The GOS (grain orientation spread) results of Ti−1300 alloy after solution treatment at the two strain rates of 200 s^−1^ and 500 s^−1^ are shown in Figure 6. 

The GOS value can reflect the number of grains that take part in the deformation. At the strain rate of 200 s^−1^, more grains with higher GOS values are near the fracture. However, at the strain rate of 500 s^−1^, the number of grains that take part in the deformation is not large. There are more gray grains with low GOS values in Figure 6b than in Figure 6a. This indicates that with the increase of the strain rate, the deformation becomes more localized, and the deformed grains tend to be located near the fracture. 

#### 3.2.3. Analysis of the Deformed Microstructure of Ti-1300 Alloy

**Figure 7:**
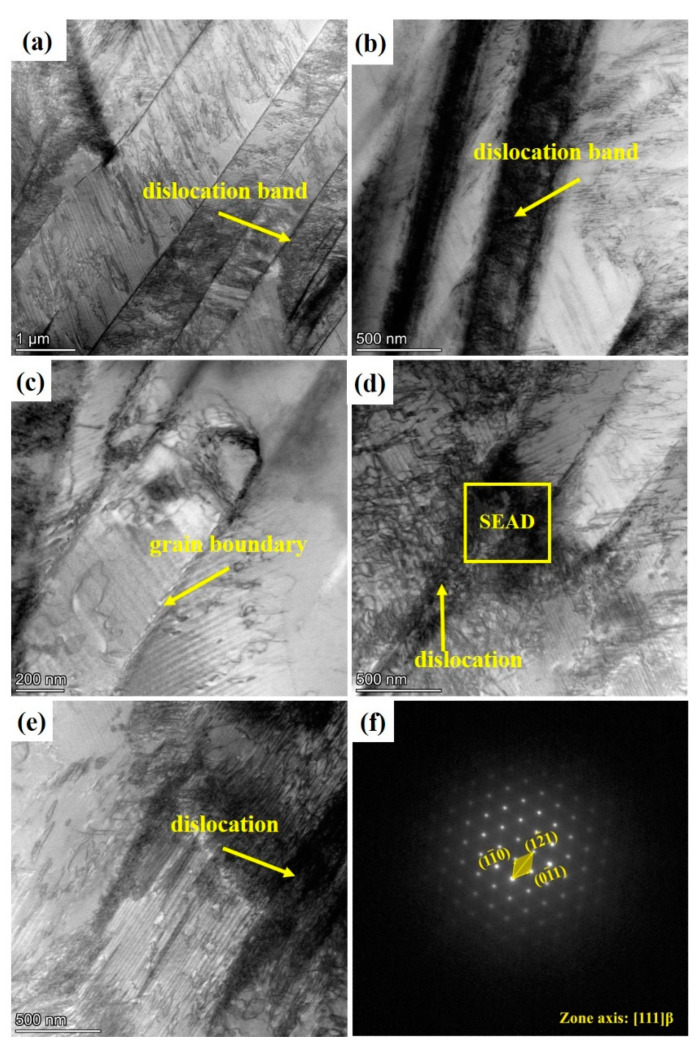
TEM pattern and diffraction spot of Ti-1300 alloy at 200 s^−1^: (**a**–**e**) TEM bright-dark field; (**f**) diffraction patterns.

Figure 7 shows the TEM images and diffraction spots of Ti-1300 alloy after high-speed tensile testing at 200 s^−1^. In Figure 7a, there are many dislocation substructures in the β grains, including dislocation bands [29] (Figure 7b). These dislocation substructures are due to the high-speed tensile testing. The grains are elongated. As the internal structure of the grains is observed, it is found that the dislocations are hindered by the grain boundaries (Figure 7c). A large number of dislocations gather near the grain boundaries and cannot break through [30,31] (Figure 7c–e). Dislocation activation forms a certain amount of slip lines, producing a fibrous substructure. It is confirmed that twinning diffraction spots are not found in the high-speed tensile microstructure in Figure 7f. 

**Figure 8:**
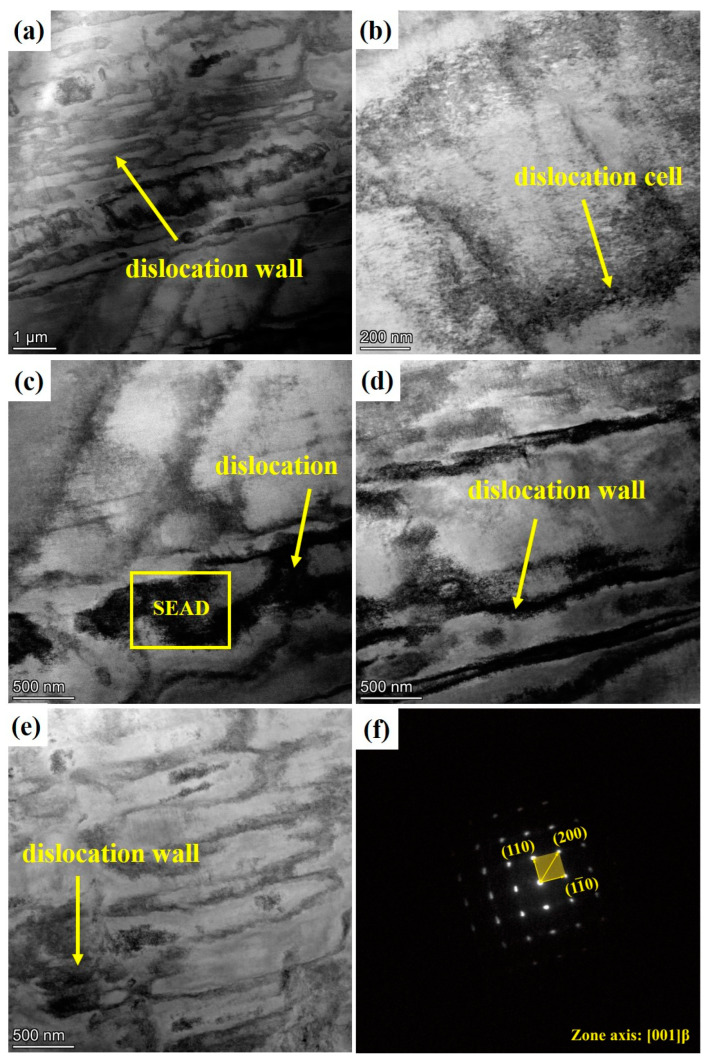
TEM pattern and diffraction spot of Ti-1300 alloy at 500 s^−1^: (**a**–**e**) TEM bright-dark field; (**f**) diffraction patterns.

No twin diffraction spots were found in Figure 8f. The results show that the dislocation slip is the main deformation behavior of the joint during tensile loading [32]. Therefore, the deformation mechanism of Ti-1300 alloy at room temperature under high-speed tensile conditions at strain rates of 200 s^−1^ and 500 s^−1^ is mainly dislocation slip. However, at 500 s^−1^, the dislocation substructures inside the grains are different to those at 200 s^−1^ (Figure 7). At the higher strain rate, the dislocation pile-up is more severe, and the dislocation cells are observed in Figure 8b,d. In theory, titanium alloys have a high stacking-fault energy [33]. Thus, it not easy for them to form dislocation pile-up. However, the increase of the strain rate is equivalent to the decrease in temperature. This causes the decrease of stacking-fault energy in this alloy system, making materials undergo dislocation pile-up more easily [34]. Thus, the differences in the dislocation substructures between the two strain rates are explained (Figure 7 and Figure 8).

### 3.3. Mechanical Properties

#### 3.3.1. Analysis of Mechanical Properties of Ti-1300 Alloy

**Figure 9:**
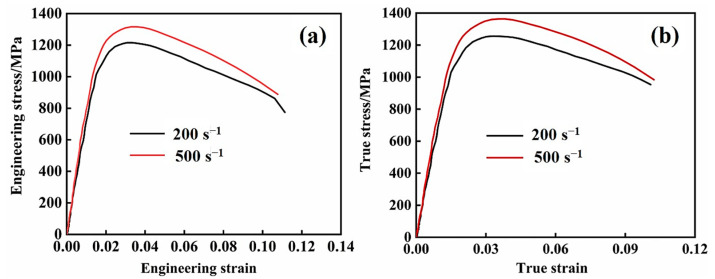
Ti-1300 alloy under high-speed tensile testing: (**a**) Stress–strain curve; (**b**) true stress–strain curve.

The engineering and true stress curves of Ti-1300 alloy at 200 s^−1^ and 500 s^−1^ strain rates are shown in Figure 9a,b. At different strain rates, the mechanical behavior is similar. At the strain rate of 500 s^−1^, the yielding strength and ultimate tensile strength (UTS) is higher, meaning that the Ti-1300 alloy has a higher sensitivity to the strain rate. The yielding strength, UTS, and elongation of Ti-1300 alloy are listed in Table 2. It is worth noting that after yielding, the UTS point is much more quickly reached on the curve. The work-hardening stage is relatively short. After the UTS point, the curves decline. Based on the above EBSD and TEM results, it seems that the mechanism of deformation only occurs through dislocation slip during high-speed tensile testing. After the solution treatment, the grains become coarse. The fact that there are not many grain boundaries explains why the work-hardening ability decreases. Compared to 200 s^−1^, the work-hardening ability in the case of 500 s^−1^ is slightly higher. This point can be noted in the difference of dislocation structures between Figure 7 and Figure 8. This limits the uniform deformation ability of the material and results in the early occurrence of necking. 

The increase of the strain rate is equivalent to the decrease in temperature. In theory, we speculate that compared to 200 s^−1^, the occurrence of dislocation climbing and cross-slip may be more difficult in the case of 500 s^−1^. Thus, the pile-up of dislocation easily happens under the condition of a high strain rate. When the deformation mechanism is the same, a higher work-hardening ability is noted at the high strain rate. However, the work-hardening ability at the two strain rates is still limited, which is due to the fact that a coarse grain structure dominates. The higher yielding strength at the high strain rate is attributed to the strain-rate hardening. The strain-rate hardening leads to the increase in the number of active dislocations per unit time.

#### 3.3.2. Tensile Fracture Analysis of Ti-1300 Alloy

**Figure 10:**
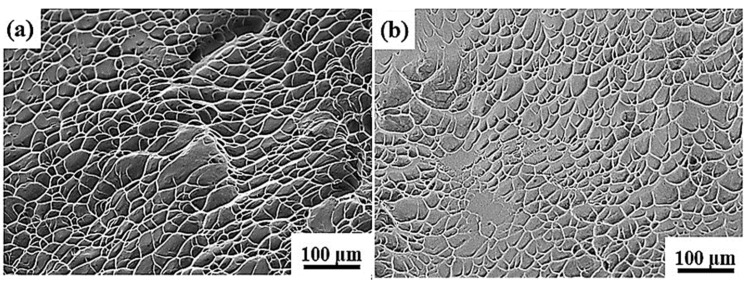
Fracture morphology of Ti-1300 alloy under high-speed tensile testing: (**a**) 200 s^−1^ and (**b**) 500 s^−1^.

In Figure 10, at the strain rates of 200 s^−1^ and 500 s^−1^, the fracture morphology of Ti-1300 alloy has the features of ductile fracture, including the dimples and tearing ridge. However, the dimples at the two strain rates are shallow, indicating that the work-hardening ability or the uniform-deformation ability is rather limited [35]. This point can be noted from Figure 9. In addition, at the strain rate of 500 s^−1^, more elongated dimples are observed at the fracture. These dimples present a parabola shape. The shape of dimples is attributed to the stress state in the local region. Thus, the elongated dimples are tearing dimples.

**Figure 11:**
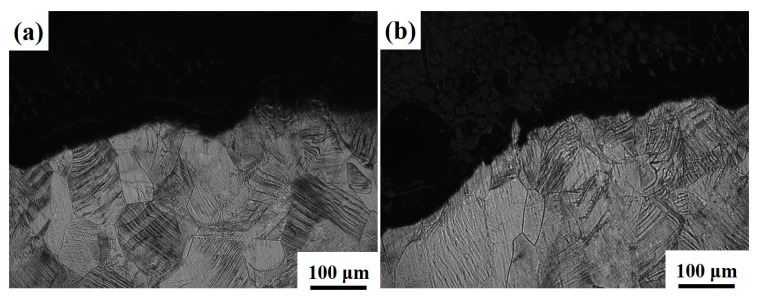
Side fracture of Ti-1300 alloy under high-speed tensile conditions: (**a**) left fracture and (**b**) right fracture. The strain rate is 200 s^−1^.

**Figure 12:**
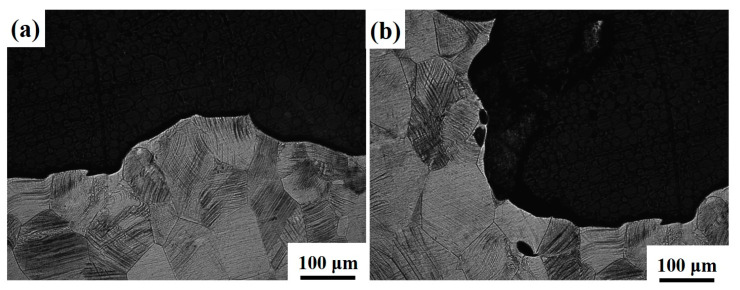
Side fracture of Ti-1300 alloy under high−speed tensile conditions: (**a**) left fracture and (**b**) right fracture. The strain rate is 500 s^−1^.

Figure 11 and Figure 12 show the side fracture of Ti-1300 alloy at 200 s^−1^ and 500 s^−1^. Similar phenomena to Figure 10 are observed. The fracture morphology presents an obvious fluctuation. At the two strain rates, ductile fracture happens. Near the fracture, many slip traces are found. In Figure 12b, voids exist close to the fracture. This may suggest that at first, micro-voids are initiated inside the material; second, these micro-voids connect with each other because of the dislocation slip; third, a crack is formed, resulting in the final fracture. 

## 4. Conclusions

(1) The single-β-phase Ti-1300 alloy does not present an obvious yielding stage. After the UTS point, an early decrease of stress indicates its limited work-hardening ability.
(2) After high-speed tensile testing at 200 s^−1^ and 500 s^−1^, only β phase is observed. The main deformation mechanism is dislocation slip.
(3) At different strain rates, the fracture mode is ductile fracture. At 500 s^−1^, a large number of tearing dimples are observed, which is due to the stress state in the local region with cracks.
(4) The connection of micro-voids induced by the dislocation slip explains the ductile fracture mode.

## Figures and Tables

**Table 1:** Geometrical parameters of high-speed tensile samples (Unit: mm).

Material	Width	Thickness	Standard Distance	Inside Diameter
Ti-1300	4.91	1.973	12.5	5
Ti-1300	4.87	1.662	12.5	5

**Table 2:** Mechanical properties of Ti-1300 alloy under high-speed tensile testing.

Strain Rate	Tensile Strength/MPa	Yield Strength/MPa	Elongation/%
200 s^−1^	1231	1107	11.3
500 s^−1^	1352	1259	10.4

## Data Availability

The data presented in this study are available on request from the corresponding author.
